# The Klippel-Trenauny Weber Syndrome: An Unusual Presentation of Hematochezia and a Review of the Literature

**DOI:** 10.4021/gr2009.05.1290

**Published:** 2009-05-20

**Authors:** Bhavesh B. Shah, Anthony Lupetin, Joseph Young, Rad Agrawal, Katie Farah

**Affiliations:** a Allegheny General Hospital, Drexel University College of Medicine Pittsburgh, Pennsylvania, USA

## To the editor:

In 2006, we met with great interest a 27 year-old male who presented to our clinic with a nine year history of intermittent painless hematochezia. This occurred approximately 8-10 times per month, and was always associated with bowel movements. He denied nausea, emesis, melena, hematemesis, constipation, abdominal pain or weight loss. Examination was remarkable for right thigh hypertrophy and large varicose veins in the right lateral thigh. His medical history was significant for a port-wine stain on his right thigh noted early in childhood, a right leg venectomy and iron deficiency anemia requiring multiple blood transfusions. In addition, he underwent laser therapy for a “bumpy” consistency of his right thigh at age 12. His workup included multiple upper endoscopies that were unremarkable, and two colonoscopies revealing “grade I internal hemorrhoids”. He had failed topical rectal suppositories in an attempt to treat his hematochezia.

**Figure 1:**
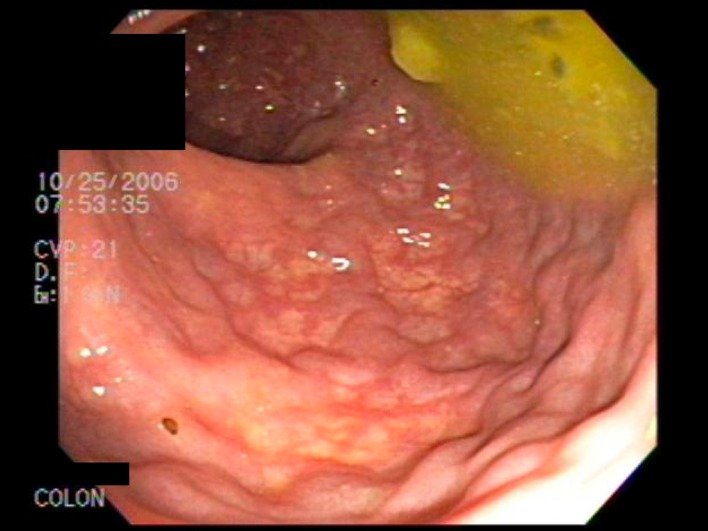
Prominent rectal veins were visualized from 10 cm to the anal verge.

**Figure 2:**
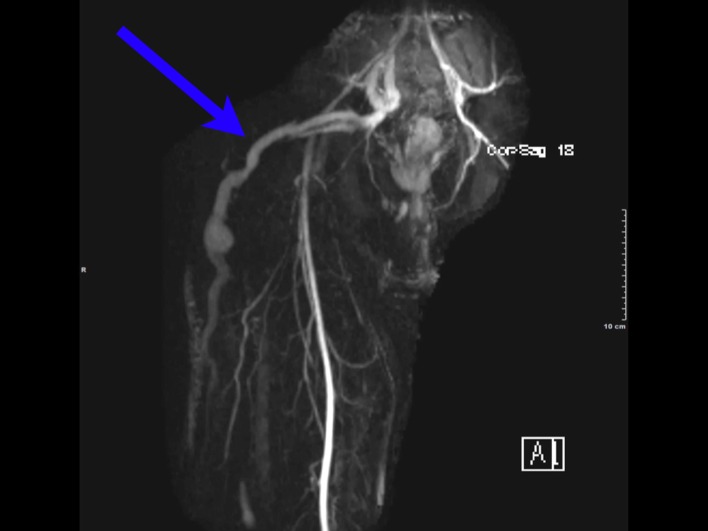
An MRI/MRV revealed an enlarged fetal lateral limb-bud vein extending into the right lateral thigh.

**Figure 3:**
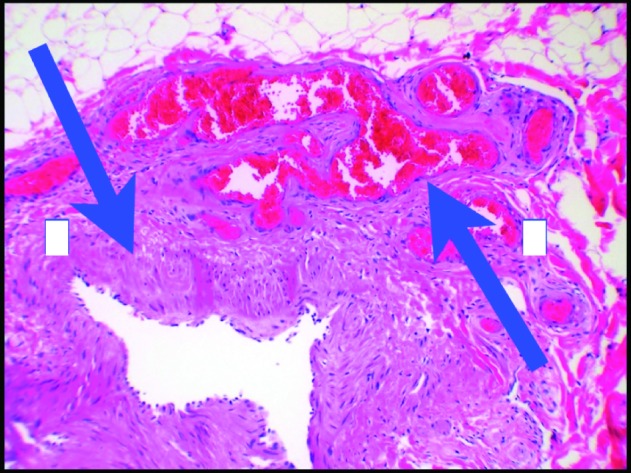
Pathology review from previous venectomy revealed arteriovenous fistulas.

Colonoscopy at our institution revealed grade I internal hemorrhoids. Prominent rectal veins were also visualized from 10 cm to the anal verge (Fig. 1). CT scan of the pelvis revealed calcified phleboliths and rectal hemangiomatosis. An MRI/MRV revealed an enlarged fetal lateral limb-bud vein extending into the right lateral thigh (Fig. 2). Vascular surgery was consulted and a right leg venogram was obtained demonstrating extensive pelvic venous collaterals and an aberrant sciatic vein. There was also communication from the common femoral vein to the venous collaterals in the knee and thigh. Pathology review from his previous venectomy revealed arteriovenous fistulas (Fig. 3). These findings were consistent with the Klippel-Trenauny Weber Syndrome (KTWS). Our patient subsequently underwent endovenous ablation of his right sciatic vein. This was followed by ultrasound-guided foam sclerotherapy. Follow-up sigmoidoscopy revealed complete ablation of his rectal varices. He has since had no further episodes of hematochezia.

The Klippel-Trenauny syndrome was originally described in 1900 as a rare, non-hereditary congenital abnormality of obscure etiology characterized by the clinical triad of soft tissue hypertrophy of an extremity, varicosities, and cutaneous hemangiomas or lymphangiomas [1]. The eponym Klippel-Trenauny Weber syndrome (KTWS) is reserved for patients with coexisting arteriovenous fistulas in the hypertrophic limb [2]. Hematochezia is an uncommon but potentially serious complication of KTWS [3]. Hematochezia can result from drainage of an overload in the posterior or compensatory venous pathways of the limb into the internal iliac vein. Several other veins, such as the rectal, pudendal, vesicular and genital veins, also drain into the internal iliac vein [3, 4]. When these veins, in this case, the rectal veins, cannot effectively drain into the dilated internal iliac vein, hematochezia occurs. Increased venous pressure during defecation and direct mucosal trauma caused by feces can aggravate recurrent hematochezia.

Endoscopy may show visible mucosal vessels or compressible nodules and bluish angiomatous submucosal lesions [3]. Mucosal ulcerations overlying the hemangiomas may sometimes be encountered and could be mistaken for inflammatory bowel disease [5]. Ligation or stripping of varicosities can worsen symptoms as new varicosities may develop and cause discomfort. In some cases, ligation of the superficial femoral or popliteal veins has resulted in shunting of blood into the dilated internal iliac vein, thereby hindering drainage of the vesical, genital and rectal veins and causing new venous malformations [6]. Control of life-threatening rectal bleeding in KTWS has been reported with angiographic embolization, placement of rectal clips, and even colectomy [3, 6, 7]. Recent developments in sclerotherapy have shown efficacy of sclerosant foam in the treatment of a variety of venous disorders [7].

We report the rare complication of chronic hematochezia secondary to the Klippel-Trenauny Weber syndrome in a young male. Prompt endoscopic recognition of rectal varices, and determination of the etiology of hematochezia is important in guiding appropriate therapy and management.
